# Individual and regional association between socioeconomic status and uncertainty stress, and life stress: a representative nationwide study of China

**DOI:** 10.1186/s12939-017-0618-7

**Published:** 2017-07-05

**Authors:** Tingzhong Yang, Xiaozhao Y Yang, Lingwei Yu, Randall R. Cottrell, Shuhan Jiang

**Affiliations:** 1 Department of Social Medicine/Center for Tobacco Control Research, Zhejiang University School of Medicine, Hangzhou, 310058 China; 2 Department Of Political Science and Sociology, Murray State University, Murray, KY 42701 USA; 3 Public Health Studies Program, University of North Carolina Wilmington, Wilmington, NC 28403 USA

**Keywords:** Socioeconomic status (SES), Life stress, Uncertainty stress, College students, Regional variance, China

## Abstract

**Background:**

Many studies have examined the association between socioeconomic status (SES) and mental stress. Uncertainty stress is a prominent aspect of mental stress. Yet no research has ever empirically analyzed the impact of SES on uncertainty stress.

**Methods:**

Students were identified through a multistage survey sampling process including 50 universities. Each student participant completed the Global Health Professions Student Survey (GHPSS) on Tobacco Control in China. Regional variables were retrieved from the National Bureau of Statistics database. Both unadjusted and adjusted methods were considered in the analyses.

**Results:**

Among the 11,942 participants, severe uncertainty stress prevalence was 19.6%, while severe life stress prevalence was 8.6%. Multilevel logistic regression showed that most SES variables were associated with uncertainty stress. Students with “operation and commercial work” as mother’s occupation and “rural or township” as family location exhibited a higher prevalence of severe uncertainty stress. Lower family income and original region gross domestic products (GDP) were also associated with higher severe uncertainty stress prevalence. However, only father’s occupation was correlated with life stress.

**Conclusions:**

Based on the literature review, this is the first empirical study examining the impact of SES on uncertainty stress in China and elsewhere in the world. Our research underscores the importance of decreasing socioeconomic inequalities in controlling excessive uncertainty stress.

## Background

A wealth of existing literature supports that social inequalities contribute to a heightened level of mental stress among the affected populace [1, 2]. Many studies have examined the association between socioeconomic status (SES) and mental stress [1–4]. Uncertainty stress refers to the stress caused by the condition of being unsure about someone or something. For example if someone was unsure about future employment status this could cause uncertainty stress. Uncertainty stress is a prominent aspect of mental stress. In general, the more uncertainty in one’s live the less comfortable one is and the more likely one is to experience stress. It is rational to hypothesize that SES should also associate to uncertainty stress. It would seem that those with lower SES would experience more uncertainty in life. Yet no research has ever empirically analyzed the impact of SES on uncertainty stress. With the rapid development of China, the emerging economic structure and the ensuing large SES differentials, a vivid sense of inequality and uncertainty among ordinary citizens exists. As economist Angus Deaton stated: “when inequality is the handmaiden of progress, we make a serious mistake if we look only at average progress. But the story is one of both growth and inequality, not just income, but health too” [5]. In this study, we hypothesized that low socioeconomic status (SES) is associated with high uncertainty stress among Chinese college students. Studies showed that uncertainty stress is a severe social and public health problem in China [6]. This study will provide evidence that socioeconomic inequalities are related to uncertainty stress. The information obtrained from this study could be helpful to inform health policy, plan prevention strategies, and design and implement appropriate, targeted interventions to help control excessive uncertainty stress.

### Hypothesis rationale

Life stress refers to the persistent daily worries in one’s life. Life stress could be related to a poor living situation, health conditions, interpersonal relationships and others [3, 6]. Most studies on mental stress and social disparities emphasize the deleterious impact of life stress. Life stressors are objective occurrences of external challenges to an individual’s coping reservoir. Uncertainty stress, on the other hand, damages mental wellbeing by challenging one’s capacity to predict and plan in such a way as to be able to act efficaciously. Compared to generic life stress, its coping requires more psychological resources because of the nature of its trigger. Uncertainty is directly related to important predictors of mental health such as self-efficacy and locus of control, which can be severely constrained when the origin of and solutions to the stress are ambiguous.

Although some scholars argue that stressors’ controllability and predictability (the lack of which leads to uncertainty stress) can sometimes be difficult to operationalize [7], a host of evidence now supports the assertion that uncertainty constitutes a powerful stressor [8]. For example, drawing upon the theories of control and defense mechanism, Mirowsky and Ross (1990) found depression to be associated with a feeling of not being in control of good and bad outcomes [9]. The stress-diathesis theory also recommends further classifications of generic stress because some stressors are desirable and controllable, while others may exert a negative or chronic influence and are harder to manage [10].

One’s social standing is a powerful determinant of the amount and quality of one’s social support, which mitigates the psychological impact of stress. Such social standing may comprise economic affluence, prestige, and ultimately the power to exercise the will [11]. Social exchange theory conceptualizes coping behaviors in response to uncertainty stress as structured by the uneven distribution of resources across social positions in a hierarchical system [12]. With fewer available material resources (money, etc.) and symbolic resources (education, prestige, etc.), a person of lower status is more likely be challenged to cope with stress. Those with fewer resources have fewer opportunities, less extensive social networks, less personal freedom, less healthy and safe work conditions, and less confidence in dealing with stress. [13]. Importantly, they have less perceived power to control their lives. The negative impact of a power differential has even been documented within non-human primates. Primates with lower power have demonstrated adverse adrenocortical, reproductive, immunological, and neurobiological functioning [14, 15]. It has been speculated that these same consequences may apply to humans in disadvantaged (less powerful) social positions [16].

In a vertically mobile social hierarchy, young adults tend to hold higher expectations for themselves regarding the development of their future career. As a result, they exert much pressure on themselves and face high expectations by the rest of society. Sorokin has argued that regardless of their objective economic standing, the upward mobile populace tend to have higher levels of stress [17]. The anticipation of any current and future threat of unknown intensity and duration constitutes a potent psychological stimulus [18]. Even when one’s socioeconomic standing has considerably improved, subjugation in a new symbolic and cultural order may still thwart self-esteem [19]. Being increasingly preoccupied with both their academic and professional development, young adults in universities experience an increasing level of uncertainty stress as college education has become a necessity for survival rather than a privilege. Students with lower SES and more challenging environments may have greater exposure to frequent and intense stress, but fewer means to manage stress [20]. This study will examine the association between SES and uncertainty stress along with life stress among college students. Given China’s regional differences in SES, region of residence might also be related to uncertainty stress and life stress. We will examine these associations at both the individual and regional level in this study.

## Methods

### Data source

This study reports individual data from students who completed the Global Health Professions Student Survey (GHPSS) on Tobacco Control in China GHPSS (Extended version). Compared to the original version, the extended version included additional health, mental stress, and behavioral items [21]. The survey was conducted between February and July 2013. A detailed description of the study methods can be found in Yang et al. [22]. Regional variables were retrieved from the National Bureau of Statistics database [23].

### Measures

#### Dependent variable

##### Stress

Life stress and uncertainty stress were measured through standard questionnaires designed by Yang and colleagues [6]. Resulting stress scores manifest acceptable validity, and have been used extensively in Chinese research [6, 24, 25]. This study also shows acceptable reliability, the Cronbach’ alpha coefficients of life stress and uncertainty stress being 0.74 and 0.79, respectively.

Life stress refers to college students’ daily worries, that are related to their life situations. The questionnaire consisted of eight items covering stressors from having “too much studying to do”, “no interest in major”, “poor study conditions”, and “little support from others”, “frustration with romantic relationship”, “financial difficulty”, “poor relationship with family members”, “poor health status among family members”. Many of these questions have been used in relevant studies [6, 24, 25]. The uncertainty stress questionnaire had 4 items which covered current life uncertainty (life is instable and cannot be controlled), social change uncertainty (uncertain about what happen in future), goals uncertainty (uncertain about how to achieve goals), and social values uncertainty (cannot follow social values). The adoption of these measurements is consulted with the literature [6, 25].

All items pertaining to measures of perceived stress were rated on a five-point scale: feeling “no stress” (0); “little stress” (1); “some stress” (2); “considerable stress” (3); and “excessive stress” (4). Not applicable items were assigned a score of zero since they provided no stress to participants. A total stress score for each questionnaire was obtained by summing up all items’ scores; the higher the total score, the greater the perceived level of stress. Consistent with prior practice, a cut-off score of 24 or more in life stress and 12 or more in uncertainty stress was classified respectively as a higher score and signified higher stress levels [6, 24, 25].

#### Demographic variables

In order to control for possible individual-level confounders, demographic questions were included on age, gender, and ethnicity.

#### Individual-level SES variables

Socioeconomic status (SES) is commonly conceptualized as the social standing or class of an individual or group. SES variables were formed on resource-based measures which assessed access to material and social assets, including income, wealth, and educational attainment [9, 18].

In this study, two individual measures of SES were included. The first one was parental occupations, recorded under three categories (Operations and commercial work; Staff and administration work; Teacher, scientific and technical work). The second measure was family income (in RMB Yuans). This variable was measured through the question: “how much was the income of each person in your family last year?” Categories ranged from less than ¥1000, ¥1000 to less than ¥2000, ¥2000 and over ¥2000 (see Table 1).

**Table 1:** Demographic characteristics of sample and related variables

Group	N	% sample	Uncertainty stress		Daily life stress	
			Prevalence (%)	UnadjustedOR	Prevalence (%)	UnadjustedOR
Age (years)						
< 20	1890	12.8	17.9	1.00	12.7	1.00
20-	2388	32.3	21.2	1.23 (0.71,2.13)	9.0	0.76 (0.56,1.05)
21-	2760	30.6	16.9	0.93 (0.59,1.47)	12.6	0.99 (0.60,1.65)
22-	2448	14.4	18.7	1.06 (0.50,2.22)	10.2	0.78 (0.43,1.41)
23-	3294	9.6	27.2	1.92 (1.02,3.06)*	13.1	1.04 (0.67,1.60)
Gender						
Male	4249	44.2	12.7	1.00	13.9	1.00
Female	7693	55.8	2.9	0.69 (0.44,1.07)	9.6	0.66 (0.48,0.89)**
Father’s occupation						
Operation and commercial work	9450	71.5	21.4	1.00	12.3	1.00
Staff and administration	1737	18.9	13.2	0.56 (0.33,0.93)*	6.3	0.48 (0.29,0.80)**
Teacher, scientificand technical work	755	9.7	18.9	1.53 (0.90,2.60)	15.5	1.85 (0.98,5.32)
Mother’s occupation						
Operation and commercial work	9591	72.3	21.0	1.00	11.7	1.00
Staff and administration	1546	16.8	16.7	0.76 (0.48,1.19)	6.6	0.54 (0.34,0.85)
Teacher, scientific and technical work	805	10.9	14.8	0.65 (0.55,0.78)**	15.5	1.59 (0.94,2.71)
Grade						
1–2	4938	60.6	20.5	1.00	12.1	1.00
3–4	6712	38.5	18.2	0.86 (0.58,1.27)	10.7	0.87 (0.52,1.49)
5-	292	0.8	19.7	0.95 (0.63,1.45)	5.8	0.64 (0.39,1.07)
Ethnicity						
Han	11,136	94.4	19.5	1.00	11.4	1.00
Minority	806	55.7	21.2	1.11 (0.67.1.83)	13.8	1.25 (0.72,2.16)
Academic major						
Medical	10,507	17.7	201.	1.00	12.0	1.00
Others	1435	82.3	19.5	0.97 (0.74,1.28)	11.4	0.95 (0.78,1.14)
Income in each person in family(RMB)						
< 10,000	1181	34.3	19.8	1.00	11.1	1.00
10,000	1273	21.7	21.0	1.65 (0.55,5.00)	14.8	1.39 (0.66,2.96)
20,000+	1932	44.0	14.4	0.68 (0.49,0.95)*	11.6	1.06 (0.47,2.38)
Regional variables						
Family location						
Rural or township	3350	59.6	19.4	1.00	10.9	1.00
County town	760	17.2	30.1	1.79 (1.07,2.99)*	18.3	1.83 (0.92,3.61)
City	898	23.2	11.6	0.55 (0.48,0.67)**	10.7	0.98 (0.49,10.7)
Original region GDP			4.55 0.1027			
<50,000	5981	51.8	923.1	1.00	12.8	1.00
50,000	359	26.3	16.8	0.68 (0.45,1.02)	8.6	0.64 (0.43,1.08)
100.000	2402	22.0	14.9	0.84 (0.76,0.92)**	11.7	0.97 (0.87,1.08)
University city GDP			1.71 0.4253			
<50,000	4055	16.1	21.3	1.00	12.1	1.00
50,000	6371	61.1	20.0	0.93 (0.57,1.52)	12.0	0.99 (0.64,1.56)
100.000	1516	22.8	17.0	0.76 (0.50,1.13)	9.6	0.78 (0.47,1.25)

*P<0.05, **P<0.01

####  Regional-level SES variables

This study also included two regional measures of SES. The first measure was the student’s family home location which was classified into three categories including city, county, and rural or township. In China, home location characteristics reflect SES inequalities between students because large differences exist between urban and rural areas, and different-level cities. The second regional measure was level of economic development. GDP per capita in the province from which the students came from (original province GDP) and the GDP per capita of the city where they were studying (university city GDP) were included. Categories were “less than 40,000,” “from 40,000 to less than 50,000,” and “50,000 and more.” The above data were obtained from the National Bureau of Statistics [23].

### Data analysis

All data were entered into a database using Microsoft Excel. The data was then imported into SAS (9.3 version) for statistical analyses. Descriptive statistics were calculated to determine the prevalence of life stress and uncertainty stress. Both unadjusted and adjusted methods were considered in the data analyses, and utilized to assess associations between the dependent and independent variables. SAS survey logistic procedures were applied in the unadjusted analysis, using the university as the clustering unit, in order to account for a within-clustering correlation, attributable to the complex sample for unadjusted analysis. Associations were confirmed through application of a multilevel logistic regression model using the SAS GlIMMIX procedure [26]. We started with the Null Model, a two-level (individual and original regions) with random intercepts in building stress multilevel logistic regression models. The constant was the sole predictor in accounting for cross-regional variation in stress. To this base, we added demographic variables and different individual and regional SES variables as fixed main effects to form several multi-level models for evaluating the impact of stress. Only variables significant in the univariate analysis for the total sample were included in the final analysis. All regional and individual variables, with categories, are listed in Tables 2 and 3. The first category for each variable served as the referent in the logistic regression analysis. First, we constructed the first model (mother’s occupation model) which included variables relating to age and mother’s occupation. The second model (family income model), the third model (family location model), and fourth model (original region GDP model) included family income, family location, and original region GDP added to model 1 respectively. These models significantly improved the fit compared with the Null Model. Model fit was assessed using −2 Res Log Pseudo-Likelihood. We assessed the significance of the random parameter variance estimates using the Wald joint t test statistic.

**Table 2:** Results of multiple level models in uncertainty stress

	Null model	Model1(mother’s occupation model)	Model2 (family income model)	Model3 (family location)	Model4 (original region GDP model)
Individual level					
Age (years)(agra)					
< 20		1.00#	1.00	1.00	1.00
20-		1.78 (0.89,3.55)	1.81 (0.85,3.84)	1.64 (1.01,2.67)*	1.82 (0.86,3.88)
21-		1.29 (0.76,2.21)	1.38 (0.91,2.09)	1.25 (0.74,2.10)	1.39 (0.92,2.10)
22-		1.05 (0.64,1.72)	1.03 (0.63,1.69)	1.04 (0.65,1.68)	1.05 (0.62,1.76)
23-		1.46 (1.02,2.16)*	1.49 (1.04,2.23)*	1.47 (1.01,2.16)*	1.62 (1.04,2.58)
Mother’s occupation					
Operation and commercial work		1.00	1.00	1.00	1.00
Staff and administration		0.77 (0.47,1.27)	0.92 (0.39,2.20)	0.78 (0.54,1.12)	0.71 (0.39,1.29)
Teacher, scientific and technical work		0.72 (0.63,0.84)**	0.33 (0.12,0.88)*	0.65 (0.55,0.77)**	0.69 (0.59,0.79)**
Income of each person in family(RMB)					
< 10,000			1.00		
10,000			0.20 (0.62,2.30)		
20,000+			0.80 (0.67,0.94)*		
Family location					
Rural or township				1.00	
County town				2.10 (1.18,3.74)	
City				0.71 (0.59,0.85)**	
Regional level					
Originalregion GDP(ogdp,22,33)					
< 50,000					1.00
50,000					0.69 (0.49,0.97)*
100.000					0.84 (0.75,0.93)**
Fixed parameters	21.15**	12.46**	6.32**	10.76**	8.43**
Random parameters between original regions	5,15**	5.18**	5.17**	5.07**	4.02**

#: OR (95% C.I)

*P<0.05, **P<0.01

**Table 3:** Results of multiple level models in life stress

	Null model	Model 1
Group	OR(95% C.I)	
Individual level		
Gender		
Male		1.00
Female		0.68 (0.53,0.93)
Father’s occupation		
Operation and commercial work		1.00
Staff and administration		0.53 (0.34,0.94)*
Teacher, scientificand technical work		1.84 (0.95,5.66)
Fixed parameters	9.13**	67.33**
Random parameters between original regions	3,45**	3.37**

*P<0.05, **P<0.01

All analyses were weighted. Weights included: (1) sampling weights, as the inverse of the probability of selection, calculated at university, and (2) post-stratification weights, calculated in relation to sex, based on estimated distributions of this characteristic from a national survey [27]. The final overall weights were computed as the product of the above two weights [26]. Unadjusted logistic regression analyses were weighted using the overall participant-level weights. The multilevel analysis was weighted using sampling in regional level, subject-level weights were used post-stratification weights, respectively [26].

## Results

Valid questionnaires were completed by 97.5% of the potential students, resulting in a sample of 11,942 students from 50 different universities.

Thirteen percent of students were less than 20 years of age, 45% were either 20 or 21 years old with the remainder of the participants being more than 21 years old. Of the study sample 44% were male and 56% were female. The majority of participants (61%) were freshmen and sophomores, and 94% of the participants were Han Chinese (see Table 1).

High levels of uncertainty stress were reported by 19.6% (95% CI: 15.9%-23.3) of students, while high levels of life stress was reported by 8.6% (95% CI: 7.2%, 10.7%) of students. The unadjusted logistic analysis showed that father’s and mother’s occupations, family income, family location, and original region GDP were associated with uncertainty stress. Life stress did not associate with any of the SES variables except father’s occupation, see Table 1. Multilevel logistic regression showed that most SES variables were associated with uncertainty stress. Students with “operation and commercial work” as mother’s occupation and “rural or township” as family location exhibited a higher prevalence of severe uncertainty stress (OR: 1.39<95% CI: 1.19, 1.59>;OR: 1.41 < 95% CI: 1.18, 1.70>). Lower family income and original region GDP were also associated with higher severe uncertainty stress. ORs were 1.25 (95% CI:1.06, 1.49) and 1.21 (95% CI:1.08,1.33). However, only father’s occupation was correlated with life stress (see Tables 2 and 3).

## Discussion

Based on the results of this study, prevalence of severe uncertainty stress was 19.6% (95% CI: 15.9%-23.3), and was significantly higher than that of life stress (8.6% (95% CI: 7.2%, 10.7%). Furthermore, prevalence of severe uncertainty stress in this population was higher (11.4% < 95% CI:8.9%,13.5%) among urban residents [6]. Prevalence of their life stress was lower (16.9% < 95% CI:13.9%,20.1%) among urban residents [6]. These results indicate that it is not just the presence of life stress that impacts Chinese college students, but even more importantly the presence of uncertainty stress.

Addressing a gap in the literature, this study confirmed that most of SES variables were negatively associated with uncertainty stress. However, such association was not observed in the life stress model. The extant psychology literature has extensively discussed how and when will stressors lead to negative outcomes in life. Specifically, specific stressors (such as the life stress measured in this study) often do not lead to mental health issues as compared to uncertainty stress, due to the latter’s nature of being difficult to engage with. Relevant to socioeconomic status, one’s disadvantage in the social and economic hierarchy may translate more dramatically into uncertainly stress than specific life stress [6]. In this study, the results support the hypothesis that SES has an important influence on uncertainty stress among Chinese students.

The correlation between SES and uncertainty stress may be explained by both risk situation exposure and individual resources. Individuals with lower SES may have greater exposure to frequent and intense uncertain situations but also have less access to rewarding or potentially beneficial situations. As a result, they are more sensitive to uncertain situations compared to those with higher SES. Moreover, low SES individuals living in harsher environmental conditions possibly maintain a smaller bank of stress reducing resources—tangible, interpersonal, and intrapersonal—to deal with uncertainly stressful events compared to their higher SES counterparts [20, 28]. Due to their lack of social resources in particular, low SES individuals may not have as much self-confidence in uncertainty situation [29].

It should be mentioned that mothers’ occupation is associated with uncertainty stress while fathers’ is not. It is plausible that in the process of natural development, especially before 13 years of age, mothers have a closer relationship with children, and exert greater influence than fathers [30]. This study also showed that older students have more uncertainty stress. This may be that when students become older they would have more worries and insecure feelings towards their prospects. Further study is encouraged to explore this field of inquiry.

It should be noted that GDP from the student’s origin place was associated with uncertainty stress, but the GDP at the university’s region was not associated with uncertainty stress in this study. Such results can be explained by the nature of college students’ financial resources--mainly dependent on families from their original region.

Studies showed that uncertainty stress is a severe social and public health problem in China [6]. While the society is changing rapidly it has shown great social inequality and anomie [30, 31], which exacerbate the feeling of uncertainty. Western culture is more receptive to change, innovation, and engaging in the unknown than Chinese culture. This receptivity enhances coping skills in the face of uncertainty. However, eastern culture, which is pronounced in China, is more conservative and prone to compliance with social rules. Generally, people influenced by eastern cultures are risk-averse, or only assume known risks. Avoidance only enhances the likelihood of high stress, nervousness, and anxiety, given that uncertainty manifests as a continuous threat that calls for resolution [32].

John Dewey captured the motivation behind uncertainty reduction as in the absence of actual certainty in the midst of a precarious and hazardous world, people cultivate all sorts of ideas that would give them the feeling of certainty [33]. The anticipation of a future threat of unknown intensity and duration constitutes a potent psychological stimulus that has an effect on the pituitary-adrenocortical system and the sympathetic-adrenal medullary system. Several studies showed that uncertainty stress is associated with severe health problems and disease [34]. Strengthening the legal and market system as well as regulating the social governance are important to help reduce uncertainty stress. Society benefits from an increased focus on the foundations of socioeconomic inequalities and efforts to reduce the deep gaps in socioeconomic status. Further, it is important to teach college students how to manage uncertainty stress. Such management should emphasize maintaining hope, learning to live with chronic uncertainty, and managing information problems [35]. Students from low SES families and regions should especially be given techniques of stress management to help them deal with their feelings of uncertainty.

### Study limitations

The cross-sectional study design is an important limitation of this study; therefore, a causal link between SES and uncertainty stress along with life stress cannot be established with this work. On the other hand, we employed a large sample, and our findings met several criteria for inferring causality, including the strength of some associations, consistent multiple SES variables, regional SES variables being used, and theory supports and plausibility of effect. Future studies need to compile longitudinal data on uncertainty and other stresses. Second, mothers’ and fathers’ occupational group, family location, and regional GDP are only crude measures of SES, more and appropriate indicators will be needed. Third, this work only focused on college students. More research needs to be done on those who are not in college and are still facing significant amounts of uncertainty stress.

## Conclusion

This study provides new evidence regarding the effects of SES on uncertainty stress and other stresses among Chinese college students. Special efforts should be made to increase focus on the foundations of socioeconomic inequalities and to reduce the deep gaps in socioeconomic status for a better control of excessive uncertainty stress. At the same time, teaching college students how to approach uncertainty and manage uncertainty stress is important.

## Abbreviations

GDP: Gross Domestic Products
GHPSS: Global Health Professions Student Survey
SES: Socioeconomic Status
